# Perinatal clinical management of a rare AB_h_ variant blood group

**DOI:** 10.1111/tme.70035

**Published:** 2025-10-23

**Authors:** Aritri Mandal, Laura Eastwood, Shane Grimsley, Louise Tilley, Clare Samuelson

**Affiliations:** ^1^ Department of Haematology Sheffield Teaching Hospitals NHS Foundation Trust Sheffield UK; ^2^ Red Cell Immunohaematology National Health Service Blood and Transplant Barnsley UK; ^3^ International Blood Group Reference Laboratory National Health Service Blood and Transplant Bristol UK; ^4^ The Department of Oncology and Metabolism The University of Sheffield, Western Bank Sheffield UK

## Abstract

**Background:**

The H antigen, precursor of the A and B blood groups, is a high‐prevalence antigen. Very few H antigen‐negative (H−) blood donors are available in the United Kingdom.

**Case Presentation:**

We present the case of a second pregnancy in a 28‐year‐old woman with the very rare AB_h_ phenotype, and the presence of anti‐H, anti‐A and anti‐B. Only H− (O_h_) and AB_h_ phenotype red cells were compatible. Given the rarity of individuals with these phenotypes, there were insufficient compatible red cell units for management of a major antepartum or postpartum haemorrhage. For this high‐risk pregnancy, meticulous multidisciplinary team (MDT) planning took place between haematologists, obstetricians, anaesthetists, hospital laboratory staff and multiple teams within National Health Service Blood and Transplant including the National Frozen Blood Bank, Rare Donor Team and Red Cell Immunohaematology Laboratories. The MDT produced a birth management plan for various eventualities where blood transfusion may have been required. This process included the creation of our hospital's first vaginal cell salvage policy. A successful routine vaginal delivery was managed without transfusion.

**Conclusion:**

This case highlights the optimal care of a woman with an extremely rare blood group in pregnancy. The principles of management described here are also more widely applicable to women in whom transfusion is contraindicated, undeliverable or declined for other reasons.

## 1 INTRODUCTION

The H antigen is a high prevalence carbohydrate antigen created by the enzyme product of the *FUT1* gene on red blood cells, and forms the precursor to A and B antigens. The H antigen is also present in body secretions, encoded by the enzyme product of the *FUT2* gene.^1^, ^2^ Individuals lacking red cell H antigen, due to rare inherited variations in *FUT1*, and who are non‐secretors due to null *FUT2* allele homozygosity, are unable to produce the A and B antigens, irrespective of the presence of ABO*A or ABO*B alleles, due to the unavailable precursor structure. Those with the resultant O_h_ phenotype form anti‐H, anti‐A, anti‐B and anti‐A,B. When there is incomplete suppression of the *FUT1* and *FUT2* genes, the H‐deficient phenotypes, A_h_, B_h_ and AB_h_, result and are even rarer than O_h_. These phenotypes are postulated to arise from an ineffective *FUT1*‐encoded enzyme that creates reduced quantities of H antigen which are rapidly and completely converted to A or B.^1^

Generally, there is no deleterious effect of these phenotypes unless blood transfusion is required. Phenotypically compatible red cells are required to prevent life‐threatening acute haemolytic transfusion reactions.^2^, ^3^ At the time of writing, the English National Frozen Blood Bank (NFBB) had three frozen units from one O_h_ blood donor and two frozen units from one AB_h_ donor, therefore availability of compatible red cell units was severely limited.

Further alloimmunisation can occur in multiply transfused or pregnant individuals due to non‐host red cell antigen exposure. Successive pregnancies can lead to additional alloantibody production, further increasing the complexity of transfusion requirements and increasing the potential for Haemolytic Disease of the Fetus and Newborn (HDFN). HDFN results from transfer of maternal antibodies via the placenta to the fetus, attaching to fetal red cell antigens resulting in haemolysis.^4^ However, ABO system antibodies are predominantly of the IgM class and typically do not cross the placenta, thereby posing a low risk of HDFN.^5^

## 2 CASE PRESENTATION

We describe the management of the second pregnancy of a 28‐year‐old woman with a rare AB_h_ phenotype with anti‐H, anti‐A and anti‐B. ABO and D grouping of the booking blood sample showed discrepant results in the forward and reverse groups when tested by BioRad® card agglutination technique (CAT) (Table 1).

**TABLE 1:** Patient ABO full group results from the blood sample taken at 8 weeks of gestation (reaction strengths graded from 0 to 4+).

Anti‐A	Anti‐B	Anti‐D	Anti‐D	A1 cells	B cells
0	1+	4+	4+	4+	2+

As the ABO group was not interpretable, the patient's red cell phenotype was tested by the National Health Service Blood and Transplant (NHSBT) reference centres including by indirect antiglobulin test (IAT) with routine and extended panels of anti‐A, anti‐B and anti‐A,B reagents. She was phenotypically H antigen negative (H‐) and determined to be a non‐secretor by gene sequencing. When tested by tube techniques, the A antigen was very weak, being detected only microscopically whilst the B antigen was of moderate strength. Anti‐H and anti‐A reacted with moderate strength, whilst the anti‐B reaction was very weak. The latter two antibodies were detected with adsorbed plasma to remove anti‐H.

The phenotype was caused by a homozygous change in *FUT2* known to cause the non‐secretor status, FUT2*01N.015/*01N.015*, and a rare homozygous c.784A>T change in *FUT1* (rs781314062; Genome Aggregation Database global frequency 0.000008).^6^ It is proposed this change in the *FUT1‐*encoded enzyme resulted in the production of only very small quantities of H antigen, all of which were rapidly converted to A and B antigens, hence the production of anti‐H. The existence of anti‐A and anti‐B, despite antigen presence, was theorised to be secondary to low levels of antigen expression, similar to anti‐A1 in individuals with A_2_ antigen positivity.

The presence of anti‐H usually indicates H‐ red cells are required for transfusion to prevent life‐threatening haemolytic transfusion reaction (HTR). Sourcing such blood is difficult in the United Kingdom. However, prior to both pregnancies, the patient's sibling (X) had previously undergone routine pre‐operative blood group testing and was discovered to have the same AB_h_ red cell phenotype as the patient and S‐. X underwent altruistic blood donation and two compatible red cell units were stored in the NFBB. If required for use, the units would require defrosting, reference laboratory cross‐match testing and transportation resulting in a time delay of ~12 h with a subsequent 72‐h shelf life. This time delay practically prohibits NFBB unit use for effective management of an unanticipated emergency such as a massive obstetric haemorrhage (MOH).

Meticulous multidisciplinary team (MDT) planning was required in assessment and minimisation of peripartum haemorrhage risk, optimisation of haemoglobin and compiling an appropriate transfusion plan. The MDT included haematologists, obstetricians, anaesthetists and NHSBT staff.

## 3 ANTEPARTUM MANAGEMENT

### 3.1 NHSBT management

The patient booking samples taken at 8 weeks of gestation were investigated for the presence of additional red cell antibodies using routine NHSBT antibody panel reagents by BioRad® indirect antiglobulin test (IAT) and enzyme IAT. Weak reactivity (1+ to 2+) was observed with all S‐ cells and stronger reactions were seen with S+ red cells. The patient's autologous control and direct antiglobulin test (DAT) were negative. The presence of anti‐H was confirmed by negative reactions with H‐ cells by IAT.

The anti‐H was adsorbed using NHSBT reagent cells. The presence of anti‐S was confirmed. All appropriate exclusions based upon the patient's red cell phenotype (Table 2) were performed; no other antibodies were detected. This added to transfusional complexity. Both anti‐H and anti‐S are considered clinically significant antibodies for HDFN.^7^ Titration of these antibodies at antenatal booking, and subsequently during pregnancy, suggested a low risk of HDFN as the titration results did not exceed 2 throughout pregnancy (Table 3).^8^

**TABLE 2:** Extended red cell phenotype as tested by red cell immunohaematology, NHSBT.

D	C	c	E	e	M	N	S	s	K	k	Fy^a^	Fy^b^	Jk^a^	Jk^b^
+	+	−	−	+	+	−	−	+	−	+	+	+	+	−

**TABLE 3:** Anti‐H and Anti‐S titration results obtained during the patient's pregnancy.

Antibody specifity	Gestation		
	8 weeks	29 weeks	34 weeks
Anti‐H titre	Neat	2	Neat
Anti‐S titre	2	Not detected	Not detected

A plan for red cell unit provision was agreed following MDT discussion with both the hospital teams and NHSBT, including the Red Cell Immunohaematology (RCI) laboratory, IBGRL and NFBB.

For emergency use, where it would not be clinically appropriate to delay transfusion to obtain NFBB compatible red cell units, the planned provision hierarchy was:

- First choice: Group B, antigen negative for: c, E, K and S
- Second choice: Group B, antigen negative for: c, E and K
- Third choice: Group O, antigen negative for: c, E, K and S
- Fourth choice: Group O, antigen negative for: c, E and K

Group B red cells, rather than group O, were selected as first and second choice despite the patient having weak anti‐B. This is because the H antigen expression of group B cells is markedly reduced compared to group O, and therefore group B cells produced a weaker in vitro reaction by IAT than group O or A cells, due to the anti‐H present in the patient's plasma.

Due to the anti‐S being undetectable at 29 and 34 weeks gestation and the relatively low number of S‐ red cell units available in the hospital transfusion laboratory (HTL), the S‐ requirement was removed from second choice but group B maintained. This contradicts normal practice because group B, S phenotype unknown red cells were considered a lower clinical risk than using group O S‐ units due to the strength of the anti‐H,^3^ and also to increase the availability of suitable red cell components.

### 3.2 Hospital outpatient management

The patient was referred to consultant‐led obstetrics clinic at 11 weeks of gestation as a high‐risk pregnancy due to her rare blood group and serological results. The patient was seen every 2 months in joint haematology‐obstetrics clinic from 14 weeks of gestation until 36 weeks of gestation. Of note, her first pregnancy and delivery had been uncomplicated, except for diet‐controlled gestational diabetes, resulting in spontaneous vaginal delivery at 35 + 5 weeks.

At 14 weeks gestation in the current pregnancy, an initial bleeding risk assessment was conducted; the patient had no prior bleeding diathesis. The patient was consequently assessed as low risk of requiring allogeneic blood transfusion during her second pregnancy and delivery. A prompt anaesthetics referral was made to ensure appropriate planning of blood provision for elective and emergency scenarios.

Appropriate antenatal patient blood management (PBM) measures to reduce blood transfusion requirement included the introduction of an oral iron preparation and folic acid to optimise haemopoiesis from 19 weeks gestation. Intravenous (IV) iron would have been recommended were oral iron not tolerated. The patient's haemoglobin (Hb) was optimised with minimal phlebotomy into paediatric blood collection tubes (PBCT) and medications were avoided which could increase bleeding risk (anticoagulants, antiplatelet agents and non‐steroidal anti‐inflammatory drugs). During the pregnancy, the lowest recorded Hb was 117 g/L, MCV 90 fL, MCH 28 Pg, Ferritin 17 μg/L at 28 weeks gestation with improvement to Hb 132 g/L post 10 weeks of oral iron supplementation. The patient underwent usual antenatal measures such as fetal ultrasound scanning (USS) and gestational diabetes monitoring. Antibody testing was conducted in accordance with British Society for Haematology (BSH) guidance.^8^

At 27 weeks gestation, consideration was given to a future planned induction of labour which would have allowed better timing of delivery; however, this would also confer an increased risk of instrumental delivery and bleeding, hence expectant management was recommended. The delivery management plan advised active management of the third stage of labour. By 36 weeks of gestation, the pregnancy remained uncomplicated, without evidence of antepartum haemorrhage, transfusion or HDFN. The delivery management plan was finalised and the blood bank staff alerted.

## 4 DELIVERY MANAGEMENT PLAN

### 4.1 Elective transfusion

For non‐emergency situations, recommendations were to prevent or manage anaemia with general PBM measures, for example, PO or IV iron. For allogeneic red cell transfusion, the NFBB would provide the two available AB_h_ units donated by X, taking into consideration the potential time delay for thawing units as outlined above.

### 4.2 Emergency transfusion

For the management of MOH, the MDT recommended early use of tranexamic acid (TXA), fresh frozen plasma, cryoprecipitate or fibrinogen concentrate. AB group plasma products would be selected. Cell salvage would be considered alongside surgical haemostatic measures, including hysterectomy as a last resort measure for life‐threatening postpartum haemorrhage.

Our hospital devised its first vaginal cell salvage policy as an alternative to allogeneic blood transfusion. This would take place in theatre in the context of MOH or retained placenta. After delivery of the neonate and amniotic fluid, excess vaginally shed blood would be suctioned into a sterile collection bag via two sterile drapes. The salvaged blood would then be processed through a leukocyte depletion filter, which has been shown to significantly reduce bacterial contamination.^9^ After liaison with microbiology colleagues, to further mitigate the bacterial contamination risk, ante‐ and intra‐partum microbiological swabs would be taken of the vagina (Group A and B streptococcus *sp*) and rectum (enterococci *sp*) to guide future antibiotic provision. Salvaged blood would undergo bacterial culture. The patient would receive salvaged blood alongside initial empirical IV cefuroxime and IV metronidazole. In addition to minimising allogenic transfusion risk, this process could potentially allow faster blood administration.

In life‐threatening MOH, the HTL would be contacted, and blood products would be provided in accordance with the prioritisation hierarchy listed above. Transfusion would take place with concurrent immunomodulatory cover; high‐dose steroids (1 g methylprednisolone or 200 mg hydrocortisone) and intravenous immunoglobulin (IVIg). In the event of further physiological deterioration, anaesthetists and/or intensivists would be contacted.

### 4.3 Neonatal management

There was a previously established low risk of HDFN associated with the maternal antibodies identified. It was recommended that fetal cord blood be tested for blood grouping, full blood count (FBC) and direct antiglobulin test (DAT). Any antepartum clinical concern for HDFN risk (serial rise in antibody titre or fetal distress on USS) would be managed by the foetomaternal unit (FMU) who would perform serial fetal Doppler USS monitoring, give consideration to intra‐uterine transfusion and further investigate neonatal haemolysis.^4^ Any neonatal transfusion would utilise O_h_ units which would be given after IAT crossmatch against the patient's plasma. HDFN occurring postpartum would be managed as usual by the paediatricians. Given the low HDFN risk, referral to FMU or paediatrics was not required in this case.

### 4.4 HTL management

**FIGURE 1:**
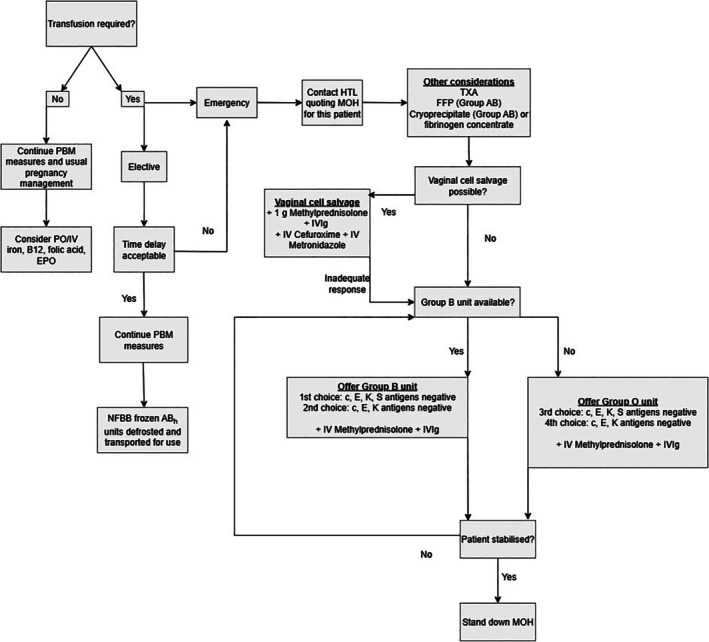
Flowchart to summarise obstetric haemorrhage plan. FFP, fresh frozen plasma; HTL, hospital transfusion laboratory; IV, intravenous; IVIg, intravenous immunoglobulins.; MOH, massive obstetric haemorrhage; NFBB, National Frozen Blood Bank; PO, per oral; TXA, tranexamic acid.

The HTL served as the central point for communication between hospital and NHSBT teams and facilitated sample transport to RCI and IBGRL. The patient's transfusion requirements were clearly documented in the HTL computer system. Perinatally, the HTL liaised with RCI to ensure the availability of suitable red cell units according to the hierarchy above and in Figure 1.

## 5 PERIPARTUM MANAGEMENT

The patient's labour started naturally at 38 weeks gestation and she successfully delivered a live healthy infant by spontaneous vaginal delivery with an estimated blood loss of 80 mL. No red cell unit nor any other transfusion was required. Her haemoglobin at delivery was 130 g/L. The infant's cord blood DAT was negative and there was no suggestion of HDFN. The patient and child remained well postpartum.

## 6 DISCUSSION

There are many learning points from this case, notably the importance of identifying difficult to transfuse patients early, conducting an assessment of their previous and current bleeding risk and performing a blood group and antibody screen. This enables better planning of procedures through preparation and reliable communication between clinical and laboratory teams. In this case, creating a delivery management plan in a timely fashion antenatally allowed the delivery to be coordinated in a controlled manner. It predicted most eventualities and allowed for better management of potential emergency and elective scenarios by providing clear protocols for clinicians less familiar with blood transfusion complexity.

The case highlights the importance of performing blood grouping and antibody screening in accordance with BSH guidance for all antenatal cases.^8^ To minimise unnecessary allogenic blood transfusion, PBM practice should be upheld with its three main pillars: optimising haematopoiesis, reducing blood loss and improving tolerance to anaemia. To optimise haematopoiesis, ensure the patient is B12, folate and iron replete and consider erythropoiesis‐stimulating agents. A merit of our case was the utilisation of iron in the absence of anaemia to maintain a normal gestational haemoglobin. Methods of reducing blood loss include: using PBCT for venepuncture, ensuring procedures are performed by the best available operator and consideration for TXA and adequately addressing haemostatic abnormalities. Restrictive transfusion thresholds should be implemented where appropriate.^10^

Other measures include platelet and plasma transfusion, for example in the context of major haemorrhage. If the scenario requires, there are more complex interventions: intra‐operative cell salvage, or as was considered in this case: vaginal cell salvage and sourcing serologically compatible blood from suitable donors via NHSBT. We also highlight the risk of additional antibody formation with successive pregnancies, which adds to transfusion complexity, but in this case, additional antibodies became undetectable as the pregnancy progressed.

The delivery management plan described allowed a planned response for various transfusion scenarios (Figure 1). For blood provision in emergencies, the best available matched blood, not necessarily fully phenotypically or genotypically matched, would suffice to maintain organ perfusion. In this setting, there would likely be insufficient time for alloantibody formation due to the high rate of blood loss. However, pre‐existing antibodies, as in this case, could still cause HTRs. In instances where serological compatibility of transfused blood cannot be assured, concomitant steroid and IVIg should be administered to mitigate the HTR risk. For elective transfusion, blood provision should account for any antigen implicated in HTR, although in the United Kingdom there is significant difficulty providing H− blood. The usual additional red cell transfusion requirements for pregnant women apply to this case, that is, K‐ and RhD compatible, CMV negative blood.^3^, ^11^ The delivery management plan and subsequent successful pregnancy outcome was the culmination of meticulous co‐operation between hospital, laboratory and NHSBT teams.

## 7 CONCLUSION

This case emphasises the importance of early identification of individuals in whom blood transfusion poses a high risk, assessing a patient's bleeding risk, performing blood grouping with antibody screening, and finding methods to reduce allogeneic transfusion need and thereby its consequences. This was a complex transfusion case; however, the principles highlighted above, including a person‐centred MDT approach, are applicable to a wide range of patients.

## AUTHOR CONTRIBUTIONS

Aritri Mandal has written the manuscript with writing and editing of laboratory and genetics aspects by Laura Eastwood, Shane Grimsley and Louise Tilley. There was oversight and editing of the manuscript by Clare Samuelson.

## CONFLICT OF INTEREST STATEMENT

The authors have no competing interests.

## PATIENT CONSENT

The patient has consented to the use of their data for the purposes of this case report, has signed a consent form approved by the journal, which has been included in the submission.

## Data Availability

The authors do not report any novel research data in this case report. Data presented here is patient‐specific and consented for use for publication. Data has been obtained from the NHSBT Rare Donor Team and International Blood Group Reference Laboratory.
